# Understanding the Electronic Structures and Absorption Properties of Porphyrin Sensitizers YD2 and YD2-o-C8 for Dye-Sensitized Solar Cells

**DOI:** 10.3390/ijms141020171

**Published:** 2013-10-10

**Authors:** Li-Heng Han, Cai-Rong Zhang, Jian-Wu Zhe, Neng-Zhi Jin, Yu-Lin Shen, Wei Wang, Ji-Jun Gong, Yu-Hong Chen, Zi-Jiang Liu

**Affiliations:** 1 Department of Applied Physics, Lanzhou University of Technology, Lanzhou 730050, Gansu, China; E-Mails: hanlh130@163.com (L.-H.H.); wangwei3057@163.com (W.W.); gongjijun@163.com (J.-J.G.); chenyh@lut.cn (Y.-H.C.); 2 State Key Laboratory of Gansu Advanced Non-ferrous Metal Materials, Lanzhou University of Technology, Lanzhou 730050, Gansu, China; 3 Gansu Computing Center, Lanzhou, Gansu 730030, China; E-Mails: zjw@gspcc.com (J.-W.Z.); jin_n_z@163.com (N.-Z.J.); shenyl@mail.gspcc.com (Y.-L.S.); 4 Department of Physics, Lanzhou City University, Lanzhou 730070, Gansu, China; E-Mail: liuzj_scu@126.com

**Keywords:** porphyrin dye sensitizers, excited states, electronic structures, density functional theory, absorption spectra

## Abstract

The electronic structures and excitation properties of dye sensitizers determine the photon-to-current conversion efficiency of dye sensitized solar cells (DSSCs). In order to understand the different performance of porphyrin dye sensitizers YD2 and YD2-o-C8 in DSSC, their geometries and electronic structures have been studied using density functional theory (DFT), and the electronic absorption properties have been investigated via time-dependent DFT (TDDFT) with polarizable continuum model for solvent effects. The geometrical parameters indicate that YD2 and YD2-o-C8 have similar conjugate length and charge transfer (CT) distance. According to the experimental spectra, the HSE06 functional in TDDFT is the most suitable functional for describing the Q and B absorption bands of porphyrins. The transition configurations and molecular orbital analysis suggest that the diarylamino groups are major chromophores for effective CT excitations (ECTE), and therefore act as electron donor in photon-induced electron injection in DSSCs. The analysis of excited states properties and the free energy changes for electron injection support that the better performance of YD2-o-C8 in DSSCs result from the more excited states with ECTE character and the larger absolute value of free energy change for electron injection.

## 1. Introduction

In recent years, dye-sensitized solar cells (DSSCs), as a novel technology for the conversion of solar energy into electricity, have attracted tremendous and continuous research interest because of easy fabrication, lower cost, and relatively higher efficiency comparing to other photovoltaic technology [1–5]. It has been found that all of the main components in DSSCs, including dye sensitizers, anode, and cathode, as well as electrolyte, can affect the photon-to-current conversion efficiency (PCE). Specifically, the dye sensitizers, which have the function of light harvesting and photon-excited electron injection, have a significant influence on the PCE [6–11]. The DSSCs applied ruthenium-complex dyes exhibit more than 10% PCE due to their broad absorption, the longer life-time of exciton, and their long-term chemical stability [4,12,13]. However, the rare resource of metal Ru and other environmental problems will limit the further development of Ru-based DSSCs for commercialization. Therefore, in order to improve the PCE and to overcome the drawback of Ru-complex sensitizers, the organic dye sensitizers, including cyanines [14–16], hemicyanines [17,18], triarylamins [8,19,20], perylenes [21–25], coumarins [26–28], porphyrins [29–34], squaraines [35–37], indoline [38,39], and azulene-based dyes [40], *etc.* were developed because of their high molar absorption coefficient, relatively simple synthetic procedure, easily modified structures and lower cost. Among them, porphyrins are viewed as one of the more promising dye sensitizers for DSSCs because porphyrin derivatives have better photosynthesis performance, strong UV-visible light absorption, and allow for easy modification/design of their structures [41,42].

The promising development of various porphyrin sensitizers to enhance the PCE was activated by over 7% PCE of porphyrin sensitized DSSCs [43,44]. During the development of porphyrin sensitizer, the PCE of DSSCs has been improved continually since the reported DSSC with 2.6% PCE was sensitized by copper chlorophyllin [45]. The further optimized DSSCs with zinc porphyrin sensitizer YD2-o-C8, co-sensitized with an organic dye Y123, using a cobalt-based electrolyte, attained PCE of 12.3% [46]. This stimulates the investigation of the further development of porphyrin sensitizers to promote the device performance of porphyrin-sensitized DSSCs. For instance, diketopyrrolopyrrole-zinc prophyrin, combining a zinc porphyrin and a dikepyrrolopyrrole fragments which display complementary absorption features into a fully conjugated push-pull systems, achieved 7.74% PCE [47,48]. Based upon analysis of the reported works on porphyrin-based dye sensitizers, it has been recognized that the intrinsic advantages of porphyrin-based dyes are their rigid molecular structures with large absorption coefficients in the visible and infrared region and their four meso and eight β reaction sites, which are available for tuning of the optical, physical, electrochemical and photovoltaic properties of porphyrins [49].

**Scheme 1:**
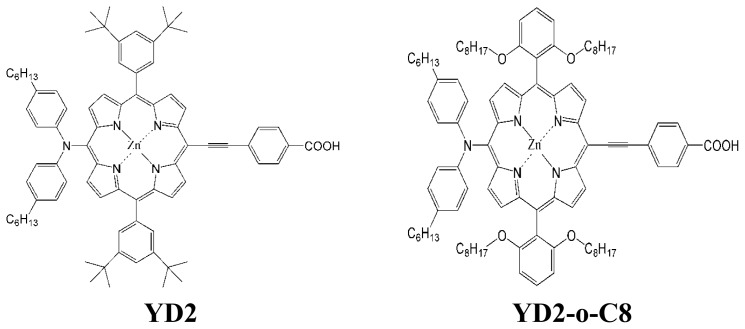
Structures of dye sensitizers YD2 and YD2-o-C8.

Further developments of the novel dye sensitizer depend on the quantitative knowledge of dye sensitizers [9,11,50]. The theoretical investigations of the physical properties of dye sensitizers are very important to disclose the relationships among the performance, structures and the properties. For instance, on the basis of density functional theory (DFT) calculations for the chemical structures and exciton binding energies of several pure organic dyes, the novel organic triphenylamine-based dye sensitizer EB-01 was designed, and over 9% of PCE was achieved by EB-01 sensitized DSSC [51]. To further design the porphyrin dye sensitizers, it should be understood why porphyrin dyes, especially YD2-o-C8, perform better than that of Ru-complex dye sensitizers, and why the dye structures and the related photophysical properties strongly influence the overall PCE. To answer these questions, it is necessary to accurately calculate electronic structures and excitation properties. However, it has been found that the suitable functional for charge transfer (CT) excited states depends on the system of dye sensitizers under study [52]. For instance, the appropriate functionals for triphenylamine and tetrahydroquinoline dyes are BHandH and CAM-B3LYP, respectively [53–56]. While for five kinds of representative dyes (L0, D4, D5, C217, and JK2), Pastore *et al.* reported that the MPW1K and CAM-B3LYP functionals represent a valuable tool of comparable accuracy to that of high level *ab initio* methods [57]. Generally, porphyrins show intense Soret bands at 400–450 nm and moderate Q bands at 500–650 nm [58]. The transition configurations of different bands involve different contribution of molecular orbitals (MOs), implying a different CT character. In this paper, YD2 [30] and YD2-o-C8 [46] were selected as representative porphyrin dye sensitizers, and their chemical structures are presented in Scheme 1. Under the framework of time dependent density functional theory (TDDFT), the suitable functional for describing CT excited states of porphyrin dyes were carefully selected based on experimental results. Based upon reliable calculations, the electronic structures and photophysical properties of YD2 and YD2-o-C8 were analyzed.

## 2. Results and Discussion

### 2.1. Geometrical Structures

**Figure 1:**
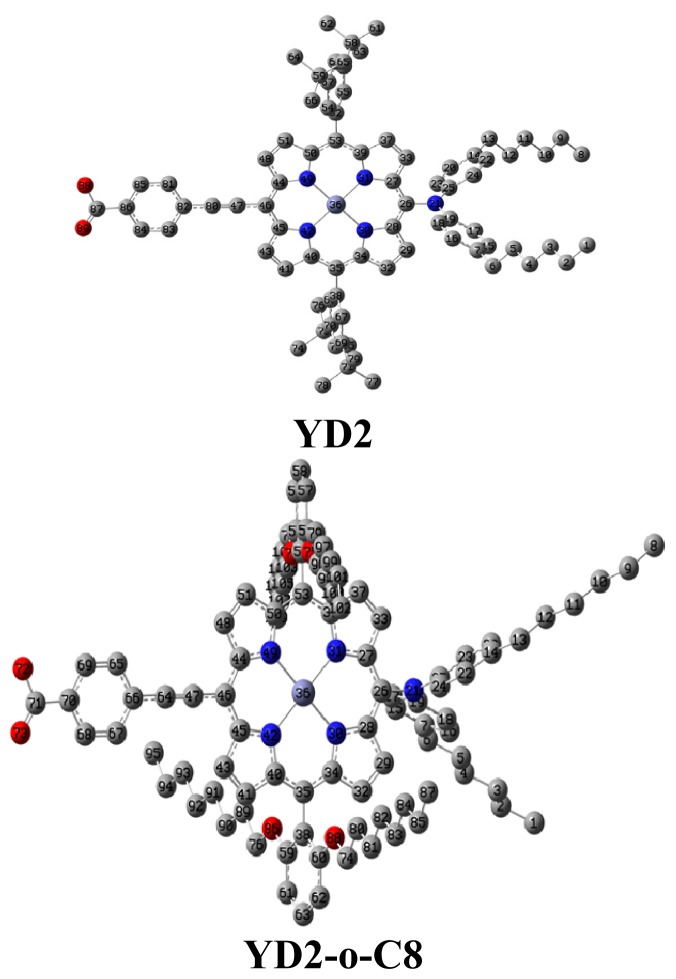
The optimized geometrical structures of YD2 and YD2-o-C8 (Hydrogen atoms have been omitted for clarity; gray circles: C; blue circles: N; red circles: O; light blue circles: Zn).

The optimized geometries of YD2 and YD2o-C8 are shown in Figure 1, where the hydrogen atoms were omitted for clarity. YD2 consists of a diarylamino group with two hexyl chains attached to the porphyrin ring acting as an electron donor, phenylethynyl group as a part of bridge, the carboxylic acid moiety as an acceptor, and the porphyrin chromophore constitutes the π bridge in this particular donor π-conjugate-bridge acceptor (D-π-A) structure [59]. The structure of YD2-o-C8 is quite similar to that of YD2. The structural feature of YD2-o-C8 involves long alkoxyl chains in the ortho-positions of the meso-phenyls, which envelope the porphyrin ring to decrease the degree of dye aggregation and to block the approach of the electrolyte to the TiO_2_ surface [60–62]. The selected geometrical parameters are listed in Tables S1 and S2 in Supplementary Information. The corresponding bond lengths, bond angles, and dihedrals are very similar. For instance, the bond lengths of Zn–N in YD2 and YD2-o-C8 are about 2.04 Å, and the bond angles of C19–N21–C25 are about 122.0°. But, the difference of dihedral C39–C53–C52–C55, which is induced by the substituting alkoxyl chains in YD2-o-C8 for tert-butyl group in YD2, is about 30° because of steric hindrance. Furthermore, the corresponding geometrical parameters of YD2 are also close to that of YD1 [63]. Therefore, the substitution at ortho-positions of the meso-phenyls has slight effects on the geometry of porphyrins framework. In addition, the quasi-coplanarity between acceptor group (carboxylic acid moiety) and conjugate bridge is favorable for intramolecular charge transfer (IMCT) [64]. The twist between diarylamino group and porphyrin cycle, which results from the orbital hybridiazation of N in diarylamino group, limits the electron donating ability [48]. The distance between N in diarylamino group and C in COOH can be defined as the conjugate length, which can describe the CT distance to some extent [56]. The conjugate lengths of YD2 and YD2-o-C8 (about 16.7 nm) indicate similar CT distance at the interface between semiconductor and dye sensitizers. Interestingly, for YD2 and YD2-o-C8, the inverse of IP optimized range-separation parameter (1/ω, 17.6 nm) is close to the conjugate lengths. The relationship between IP optimized range-separation parameter and conjugate length is similar to that of oligoacenes, polyenes, and oligothiophenes [65].

### 2.2. Functional Selection for TDDFT

**Figure 2:**
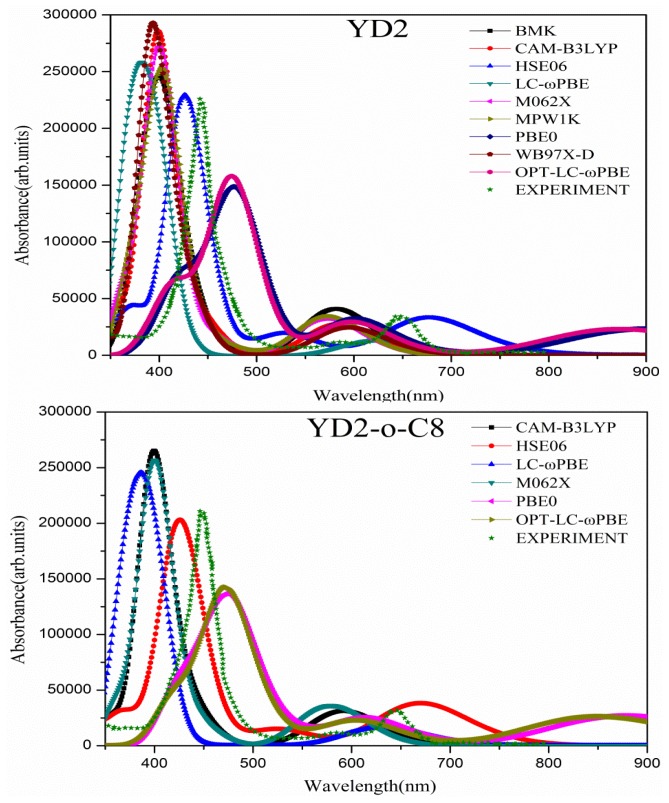
The simulated absorption spectra for YD2 and YD2-o-C8 dyes based upon TDDFT results calculated with different functionals. The experimental measured curves [46] are also included.

The UV-vis absorption spectra is the most convenient available experimental data, which can be adopted as the benchmark for selection of suitable TDDFT functional for excited properties of dye sensitizers. For porphyrins, the first absorption band was usually denoted as Q band, while the transitions with the highest oscillator strengths were designated as the B-bands [63]. The calculated absorption λ_max_ (nm/eV), absolute errors (AE, in nm/eV), arithmetic mean absolute errors (AMAE, in nm/eV), and mean square error σ (in eV) of YD2 were listed in Table 1. The data indicates that the most accurate functional for B and Q bands are HSE06 and LC-ωPBE, respectively. Averagely, HSE06 generate the smallest AMAE for these bands (about 0.11 eV) and σ (about 0.11 eV) then the smaller AMAE of LC-ωPBE is about 0.19 eV. The calculations with MPW1K, BMK, and ωB97XD functionals for YD2-o-C8 were not conducted because their AE were large. The calculated results for YD2-o-C8 were listed in Table 2. The data indicates that the most accurate functional for B and Q bands are OPT-LC-ωPBE and LC-ωPBE, respectively. However, again, HSE06 generate the smallest AMAE (about 0.13 eV) and σ (about 0.14 eV). Though LC-ωPBE generates the smallest AE for Q band, the AE for B band is quite remarkable. The simulated spectra are presented in Figure 2. Apparently, the experimental spectra’s character, including λ_max_ and line shape (relative strength), is well reproduced by the calculation with HSE06 functional. So the HSE06 functional is the most suitable functional for YD2 and YD2-o-C8 to describe their excitations.

### 2.3. Electronic Structures

**Figure 3:**
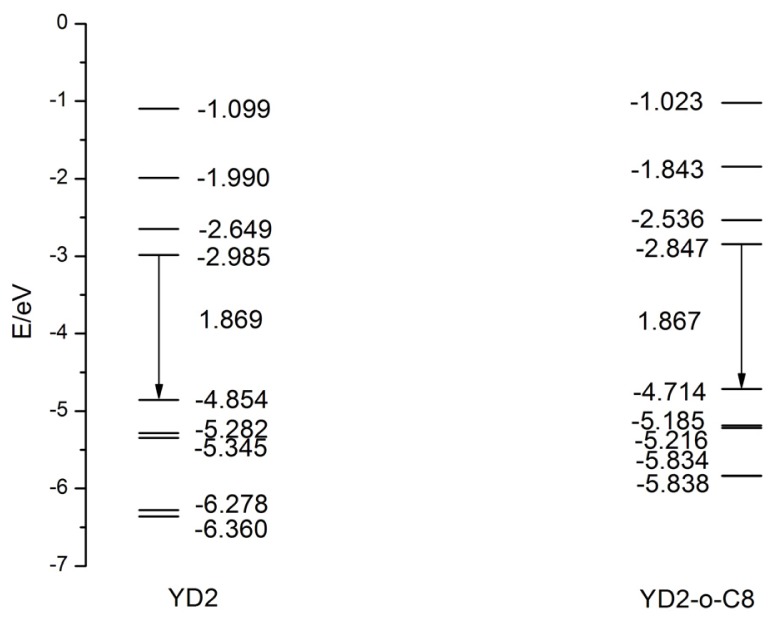
The calculated frontier molecular orbitals energies and HOMO-LUMO gap at the HSE06/6-31G (d,p) level in THF solvent.

**Figure 4:**
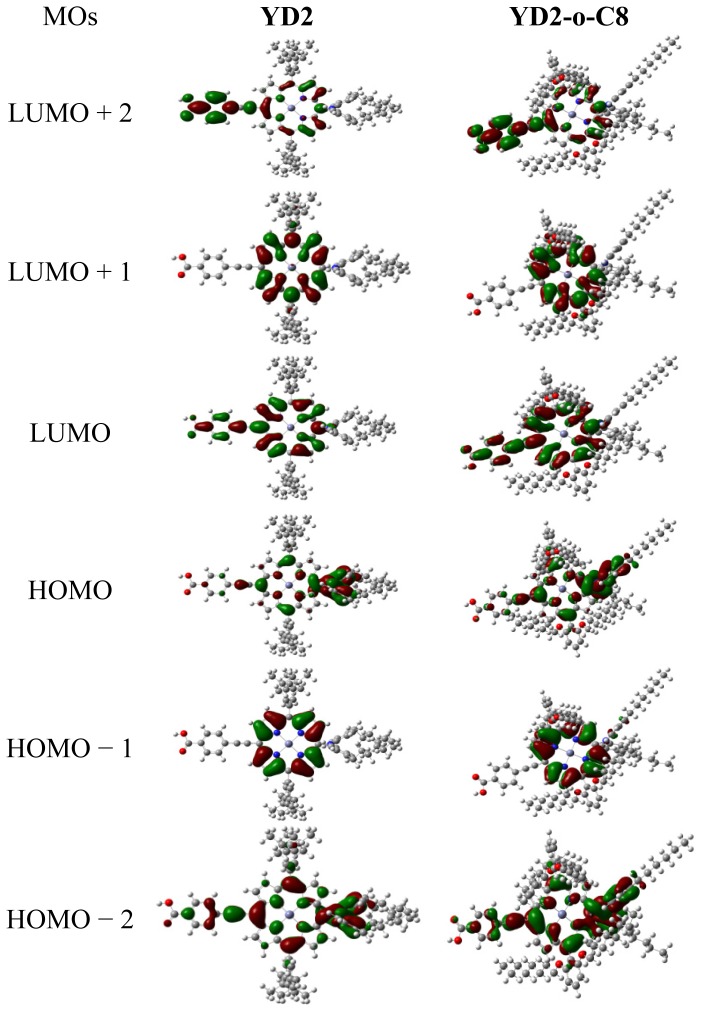
Isodensity plots (isodensity contour = 0.02 a.u.) of the frontier orbitals of the dye YD2 and YD2-o-C8.

The highest occupied molecular orbital (HOMO) and the lowest unoccupied molecular orbital (LUMO) energies of better dye sensitizers are required to locate suitable values for matching the conduction band edge of semiconductor and redox potential of electrolyte in DSSCs. The HOMO level corresponds to the ground state oxidation potential (GSOP) of dye sensitizer [66], and the larger GSOP increase the driving force for the reduction of oxidized dye [67]. The HSE06 functional calculated frontier MO eigenvalues and the HOMO-LUMO gaps, as well as isodensity plots of frontier MOs of YD2 and YD2-o-C8 in THF are shown in Figures 3 and 4, respectively. For ZnTPP, the HOMO and HOMO − 1 have a_2u_ and a_1u_ symmetry, respectively, while the degenerate LUMOs (LUMO, LUMO + 1, and LUMO + 2) have e_g_ symmetry. For YD2, the eigenvalue of HOMO − 4 is very close to that of HOMO − 3, only about 0.08 eV difference. The HOMO − 2, lying at about −5.35 eV, mixes the MOs of diarylamino and phenylethynyl groups with porphyrin a_2u_ orbital, which has electron density that located on the bound α position. The HOMO − 1, which is about 0.07 eV above HOMO − 2, remains relatively unperturbed local porphyrin a_1u_ orbital due to the mismatching of symmetry, and this is different with the mixed MOs of diarylamino and phenylethynyl groups with porphyrin (B3LYP/6-31G(d) results [46]). The difference can be understood from the different DFT functional [68]. The HOMO, locating at about −4.85 eV, is also mixed orbital which mainly contributed by diarylamino and porphyrin a_2u_ orbital. The LUMO and LUMO + 2 mix the MOs of phenylethynyl carboxylic acid with porphyrin e_g_ orbital. But the LUMO + 2 contains more phenylethynyl carboxylic acid derived orbital character compared with the porphyrin based e_g_ orbital. Conversely, the LUMO contains a great contribution of the e_g_ derived orbital than from the phenylethynyl carboxylic acid MO. The LUMO + 1, localizing on porphyrin, keeps the unperturbed e_g_ character. The frontier MOs characters of YD2-o-C8 are quite similar to that of YD2. The HOMO − 1 and HOMO − 2, the HOMO − 3 and HOMO − 4 of YD2-o-C8 are quasi-degenerate, respectively. Furthermore, the substitution of tert-butyl groups in YD2 by octyloxy groups in YD2o-C8 increase HOMO about 0.140 eV and LUMO about 0.138 eV and thus reduce the HOMO-LUMO gap about 0.002 eV, that is to say, introducing octyloxy groups which have long alkyl chains decrease the energy gap very slightly. The increase of orbital energies of YD2-o-C8 may be rationalized by considering the electron-donor character of octyloxy groups.

YD2 has a lower HOMO than YD2-o-C8, which results in a larger free energy change for dye regeneration (Δ*G^regen^*) since the Δ*G^regen^* can be determined by dye oxidation potential and redox potential of electrolyte [64]. Also, it should be noted that the tert-butyl groups in YD2 and the octyloxy groups in YD2o-C8 are independent of the frontier MOs. That’s why the dyes have almost same HOMO-LUMO gap. The role of the tert-butyl and the octyloxy groups might be to reduce the dye aggregation and block the approach of the electrolyte to the TiO_2_ surface, therefore control the regeneration site of the dyes and suppress charge recombination.

### 2.4. Absorption Properties

The absorption properties determine the capability of harvesting photons, and thus affect the performance of dye sensitizers in DSSCs. The absorption spectra of YD2 and YD2-o-C8 in UV-vis region were measured in THF solution. The experimental and the HSE06 functional calculated results are listed in Table 3, which includes the major singlet transition configurations with coefficients larger than 10% and the oscillator strength larger than 0.1, while data calculated with other functionals are listed in Tables S3 and S4 in Supplementary Information. The spectral similarity of YD2 and YD2-o-C8 indicates that the substitution of tert-butyl groups in YD2 by octyloxy groups in YD2o-C8 very slightly affect the absorption properties. This can be understood from the MO analysis.

The transition configurations in Table 3 and MOs in Figure 4 indicate that the overlaps between initial states to final states are highly coupled to the porphyrin ring. This again emphasizes the role of π-conjugated linker. The larger overlap based porphyrin generates larger oscillator strength and stronger absorption bands than that of D-π-A organics dyes containing other conjugated linkers, such as thiophene. To investigate the excited states properties, it is necessary to distinguish between local excitation (LE), effective charge transfer excitation (ECTE) *i.e.*, CT excitation and extending electron distribution to anchor group of dye, ineffective CT excitation (ICTE) *i.e.*, a CT excitation where the electron distribution does not extend to the anchor group of the dye. The transitions of the dyes that involve HOMO − 1→LUMO + 1 are typical LE since the related MOs are localized in porphyrin. The relocations of the HOMOs and LUMOs in transition configurations support that the excitations have ECTE character, for instance, the transitions involving HOMO→LUMO, HOMO→LUMO + 2, *etc.* However, the final states in transitions involving LUMO and LUMO + 2 for the dyes are ECTE, while the final states in transitions involve LUMO + 1 are ICTE. Apparently, the transitions that related to HOMO − 2→LUMO + 1 are ICTE. The further transition configuration and MO analysis indicates that the diarylamino groups are major chromophores for ECTE, and actas electron donor in photon-induced electron injection in DSSCs. It should be noticed that the S8 state of YD2-o-C8 involving HOMO − 4→LUMO is ECTE because HOMO − 4 mainly contributed by meso-phenyls in the ortho-positions and O in alkoxyl chains. Therefore, the meso-phenyls in the ortho-positions and O in alkoxyl chains in YD2-o-C8 also participate in donating electrons.

Based upon the above analysis, the number of excited states with ECTE character of YD2-o-C8 is larger than that of YD2. More ECTE is favorable for more charges of CT in DSSCs. Under the same experimental condition, the open-circuit voltage (V_oc_) and short-circuit current density (J_sc_) of YD2-o-C8 is larger than that of YD2 [46].

The photo-induced electron injection in DSSCs can be viewed as a CT process. In terms of Marcus theory for electron transfer [69], the CT rate constants can be influenced by the free energy change. The free energy change for electron injection (Δ*G^inject^*) affects the electron injection rate and therefore the J_sc_ and V_oc_ in DSSCs. Therefore, Δ*G^inject^* can be viewed as the electron injection driving force [55]. The computational methods for Δ*G^inject^* and the oxidation potential of excited state with ECTE character ( $E_{OX}^{dye*}$) are adopted using Preat’s method [53]. For the case of electron injection, which occurs from the unrelaxed excited states of dye, the Δ*G^inject^* can be calculated as $\Delta G^{inject}=E_{OX}^{dye*}-E_{CB}^{SC}$. The $E_{OX}^{dye*}$ is the oxidation potential of the dye in excited states and $E_{CB}^{SC}$ is the conduction band of the semiconductor in electrode. The reported conduction band edge for TiO_2_ is 4.0 eV [70]. The $E_{OX}^{dye*}$ can be calculated as $E_{OX}^{dye*}=E_{OX}^{dye}-\lambda_{\text{max}}$, where $E_{OX}^{dye}$ is the oxidation potential of the ground state and λ_max_ is the absorption maximum with ECTE character. Due to the molecular sizes of dye sensitizers in this work, the $E_{OX}^{dye}$ is approximated by applying the Koopmann’s theorem where it uses the absolute value of HOMO energy [63]. Usually, there are three absorption bands for metalloporphyrins in UV/Vis region. The prominent band to the red edge of B band was assigned as the T band [71], and the absorbance of T band is quite weaker than that of Q and B bands. The calculated $E_{OX}^{dye*}$ and Δ*G^inject^* of Q, T, and B bands for all the dyes are listed in Table 4.

The calculated $E_{OX}^{dye*}$ of Q, T, and B bands for YD2-o-C8 are smaller than that of YD2 due to its smaller ground state oxidized potential. The results in Table 4 also indicate that the Δ*G^inject^* of the dyes are negative, which means that the dye excited state with ECTE character lies above the TiO_2_ conduction band edge. The data also suggest that the substitution of tert-butyl groups in YD2 by octyloxy groups in YD2o-C8 increase the absolute value of Δ*G^inject^*. The larger absolute value of Δ*G^inject^* is favorable for fast electron injection, and thus for improving J_sc_ and V_oc_.

## 3. Computational Methods

The computations of the geometries and electronic structures for dye sensitizers were performed without any symmetry constraints using DFT in the Gaussian09 package [72]. The polarized split-valence 6-31G(d,p) basis sets are sufficient for calculating the excitation of organic dyes [73], and introducing additional diffuse functions in basis sets generate negligible effects on the electron density and hence on the accuracy of DFT and TDDFT results [57]. Therefore, the 6-31G(d,p) basis sets were adopted in the present work. The commonly referred B3LYP functional, which combines of Becke’s 3-parameter exchange functional [74] and the correlation functional from Lee, Yang, and Parr [75], was adopted during the geometry optimization in the phase of gas and solvent because B3LYP is known to provide molecular geometries in good comparison to experiment [76]. The solvent effects were considered using a non-equilibrium version of the polarizable continuum model (PCM) [77] method. The electronic excitations were investigated using TDDFT method. Generally, for the dye sensitizers with good performance in DSSC, the electronic excitations are CT processes. However, the TDDFT calculations with conventional functionals, such as B3LYP, poorly reproduce CT excitations energies. In order to select suitable functional for the reliable description of excited properties of dye sensitizers, TDDFT were performed using different functionals, including the hybrid functionals PBE0 (25% of Hartree-Fock exchange) [78–80], MPW1K (42% of Hartree-Fock exchange) [81], BMK (inclusion of the kinetic energy density together with a large value of the exact exchange mixing coefficient) [82], M062X (a high nonlocality functional with double the amount of nonlocal exchange) [83], HSE06 (introducing an efficient screening technique to take advantage of the fast spatial decay of the short range Hartree-Fock exchange used in the Heyd-Scuseria-Ernzerhof screened Coulomb hybrid density functional) [84–88], and the long range corrected hybrid functional (the exchange term in the Kohn-Sham energy functional split into long-range and short-range terms) CAM-B3LYP [89], LC-ωPBE [90–93], ωB97X-D [94] with default range separation parameter, as well as LC-ωPBE with optimized range-separation parameter (denoted as OPT-LC-ωPBE). The system-dependent optimal range-separation parameter is determined by “IP tuning” procedure [65,95,96], minimizing the difference between the highest occupied orbital eigenvalue ɛ_HOMO_ and the computed ionization potential (IP) for ω,

(1) $$\delta_{IP}(\omega )=\left|{ɛ}_{HOMO}^{\omega }-(E_{n}(\omega )-E_{c}(\omega ))\right|$$

Here, *E_n_* and *E_c_* are total energies of neutral and cation states, respectively. The results are shown in Figure S1 in Supplementary Information (SI). Apparently, the optimized ω for YD2 and YD2-o-C8 dyes are about 0.03 Bohr^−1^. By comparing the calculated results with experimental absorption data, the suitable functional was selected. The electronic structures and photophysical properties of YD2 and YD2-o-C8 were analyzed based upon the calculated results with the selected functional. Simulation of absorption spectra was performed using Gaussian peaks with full width at half maximum of 0.15 eV.

## 4. Conclusions

In this work, the geometries, electronic structures, and excited states properties of porphyrin dye sensitizers YD2 and YD2-o-C8 have been studied using DFT and TDDFT calculations. The calculated geometrical parameters indicate that YD2 and YD2-o-C8 have similar conjugate lengths and CT distance. According to the experimental spectra, the HSE06 functional in TDDFT is the most suitable functional for describing Q and B bands of phorphyrins. The data of electronic structures suggest that the substitution of tert-butyl groups in YD2 by octyloxy groups in YD2-o-C8 slightly affects HOMO-LUMO gap. In addition, the slightly lower HOMO of YD2, comparing that of YD2-o-C8, results in a large free energy change for dye regeneration, which is favorable for fast dye regeneration. Furthermore, the independence of the tert-butyl groups in YD2 and the octyloxy groups in YD2o-C8 on the frontier MOs suggest that the role of the tert-butyl and the octyloxy groups might be to reduce the dye aggregation and block the approach of the electrolyte to the TiO_2_ surface. The transition configuration and MO analysis supports that the diarylamino groups are major chromophores for ECTE, and act as the electron donor in photon-induced electron injection in DSSCs. Compared with the performance of YD2, the better performance of YD2-o-C8 results from the more excited states with ECTE character and the larger absolute value of Δ*G^inject^*.

## Supplementary Information



## Figures and Tables

**Table 1:** The calculated absorption λ_max_ (in nm/eV) and the absolute errors (AE, in nm/eV) of B and Q bands for YD2 with different functionals in TDDFT, the arithmetic mean absolute errors (AMAE, in nm/eV) and mean square error σ (in eV) are also listed.

Functionals	λ_max_		AE		AMAE	σ
	
	B	Q	B	Q		
CAM-B3LYP	397/3.12	585/2.12	46/0.32	61/0.20	53.5/0.26	0.267
M062X	397/3.12	573/2.17	46/0.32	73/0.25	59.5/0.29	0.287
PBE0	481/2.58	902/1.37	38/0.22	256/0.55	147/0.39	0.477
BMK	403/3.08	582/2.13	40/0.28	64/0.21	52/0.25	0.247
ωB97XD	391/3.17	595/2.08	52/0.37	51/0.16	51.5/0.27	0.285
MPW1K	401/3.09	575/2.16	42/0.29	71/0.24	56.5/0.27	0.266
LC-ωPBE	399/3.11	628/1.98	44/0.31	18/0.07	31/0.19	0.225
OPT-LC-ωPBE	475/2.61	874/1.42	32/0.19	228/0.50	130/0.35	0.378
HSE06	423/2.93	677/1.83	20/0.13	31/0.09	25.5/0.11	0.112
Experiment	443/2.80	646/1.92	–	–	–	–

**Table 2:** The calculated absorption λ_max_ (nm/eV) and the absolute errors (AE, in nm/eV) of B and Q bands for YD2-o-C8 with different functionals in TDDFT, the arithmetic mean absolute errors (AMAE, in nm/eV) and mean square error σ (in eV) are also listed.

Functionals	λ_max_		AE		AMAE	σ
	
	B	Q	B	Q		
CAM-B3LYP	397/3.12	592/2.09	51/0.35	53/0.17	52/0.26	0.275
M062X	397/3.12	579/2.14	51/0.35	66/0.22	68.5/0.29	0.292
PBE0	477/2.60	879/1.41	29/0.17	234/0.51	131.5/0.34	0.380
LC-ωPBE	399/3.11	632/1.96	49/0.34	17/0.04	33/0.19	0.242
OPT-LC-ωPBE	472/2.63	853/1.45	24/0.14	208/0.47	116/0.31	0.347
HSE06	420/2.95	671/1.85	28/0.18	26/0.07	27/0.13	0.137
Experiment	448/2.77	645/1.92	–	–	–	–

**Table 3:** The experimental spectra data and the HSE06 functional calculated excitation energies (eV), wavelength (nm), oscillator strengths (f) and major transition configurations with coefficients larger than 10% in UV-vis region for YD2 and YD2-o-C8 in THF.

Dyes	States	Major transition configurations	E (nm/eV)	*f*	Experimental λ_max_/nm (ɛ/10^3^ M^−1^ cm^−1^)
YD2	S_1_	H→L (94%)	677/1.83	0.3660	646 (34)
	S_3_	H − 2→L (78%); H − 1→L + 1 (21%)	537/2.31	0.1294	586 (11)
	S_5_	H→L + 2 (73%); H − 1→L + 1 (18%)	443/2.80	0.7025	
	S_6_	H − 2→L + 1 (49%); H − 1→L (25%); H − 1→L + 2 (10%)	423/2.93	0.8193	
	S_7_	H − 1→L + 1 (39%); H →L + 2 (21%); H − 2→L (11%)	423/2.93	1.1253	443 (227)

YD2-o-C8	S_1_	H→L (93%)	671/1.85	0.4237	645 (31)
	S_3_	H − 2→L (78%); H − 1→L + 1 (17%)	535/2.32	0.1058	581 (12)
	S_5_	H→L + 2 (70%); H − 1→L + 1 (21%)	442/2.80	0.7339	
	S_6_	H − 2→L + 1 (38%); H − 1→L (24%); H − 4→L (14%)	426/2.91	0.5621	
	S_8_	H − 4→L (76%)	421/2.94	0.1303	
	S_9_	H − 1→L + 1 (42%); H→L + 2 (25%)	420/2.95	1.0332	448 (212)

**Table 4:** The calculated excited states oxidized potential ( $E_{OX}^{dye*}$, in eV) and free energy change for electron injection (Δ*G^inject^*, in eV) of Q, T, and B absorption bands for YD2 and YD2-o-C8 dyes.

Dyes	$E_{OX}^{dye*}$			Δ*G^inject^*		
	
	Q	T	B	Q	T	B
YD2	3.02	2.54	1.92	−0.98	−1.46	−2.08
YD2-o-C8	2.86	2.39	1.76	−1.14	−1.61	−2.24
