# Supplementary material for: Understanding the Electronic Structures and Absorption Properties of Porphyrin Sensitizers YD2 and YD2-o-C8 for Dye-Sensitized Solar Cells
Source: Int J Mol Sci. 2013 Oct 10;14(10):20171–88. doi: 10.3390/ijms141020171 (PMC3821609; doi:10.3390/ijms141020171)
Supplement: Supplementary file 1 [file ijms-14-20171-s001.pdf]

Supplementary Information

Table S1. The selected bond lengths (in Å), bond angles (in degree) and dihedrals (in degree) of the dye YD2. (B3LYP/6-31g(d,p), gas phase).

Definition	Values	Definition	Values	Definition	Values
19–21	1.42	19–21–25	122.0	19–21–26–28	−71.2
21–25	1.42	25–21–26	118.9	25–21–26–27	−71.1
21–26	1.43	19–21–26	119.1	28–30–36–49	−88.6
30–36	2.04	30–36–31	90.2	27–31–36–42	−92.8
31–36	2.04	30–36–42	89.8	34–35–38–68	−72.3
42–36	2.04	31–36–49	89.8	39–53–52–55	67.0
49–36	2.04	42–36–49	90.2	40–35–38–67	−72.7
46–47	1.42	31–36–42	179.1	50–53–52–54	66.8
47–80	1.22	30–36–49	179.2	43–45–46–47	1.2
80–82	1.42	34–35–38	117.6	48–44–46–47	−1.8
86–87	1.48	39–53–52	117.6	46–47–80–82	−75.5
87–88	1.36	45–46–47	117.5	47–80–82–83	−2.6
87–89	1.22	86–87–89	125.0	47–86–82–81	117.4
35–38	1.50	89–87–88	121.9	84–86–87–89	0.0
53–52	1.50	86–87–88	113.1	85–86–87–88	0.0

Table S2. The selected bond lengths (in Å), bond angles (in degree) and dihedrals (in degree) of the dye YD2-o-C8. (B3LYP/6-31g(d,p), gas phase).

Definition	Values	Definition	Values	Definition	Values
19–21	1.42	19–21–25	122.0	19–21–26–28	71.6
21–25	1.42	19–21–56	119.1	25–21–26–27	71.1
21–26	1.44	25–21–26	118.8	28–30–36–49	89.9
30–36	2.04	30–36–31	90.0	27–31–36–42	92.3
31–36	2.04	30–36–42	90.0	34–35–38–59	−86.5
42–36	2.04	31–36–49	90.0	39–53–52–55	97.7
49–36	2.04	42–36–49	90.0	40–35–38–60	−85.9
46–47	1.42	31–36–42	179.2	50–53–52–54	97.9
47–64	1.22	30–36–49	179.3	43–45–46–47	0.5
64–66	1.42	34–35–38	117.5	48–44–46–47	0.4
70–71	1.48	39–53–52	117.4	46–47–64–66	−12.8
71–72	1.36	45–46–47	117.5	47–64–66–65	114.4
71–73	1.22	70–71–73	125.1	47–64–66–67	−38.6
35–38	1.50	73–71–72	121.8	68–70–71–73	−0.1
53–52	1.50	70–71–72	113.2	69–70–71–72	−0.1

Table S3. The different functional calculated excitation energies (eV), wavelength (nm), oscillator strengths (f) and major transition configurations with coefficients larger than 10% of three stronger absorption bands in UV-vis region for YD2 in THF.

Functional	States	Major transition configurations	E(nm/eV)	*f*
CAM-B3LYP	S_1	H − 1→L + 1 (25%); H→L (69%)	585/2.12	0.3012
	S_3	H − 2→L (51%); H − 1→L + 1 (24%); H→L (16%)	443/2.80	0.3372
	S_5	H − 2→L (35%); H − 1→L + 1 (45%); H→L (10%)	397/3.13	2.0155
M062X	S_1	H − 1→L + 1 (21%); H→L (75%)	573/2.17	0.3551
	S_3	H − 2→L (58%); H − 1→L + 1 (23%); H→L (11%)	452/2.75	0.1824
	S_5	H − 2→L (31%); H − 1→L + 1 (50%); H→L (11%)	397/3.12	2.1369
PBE0	S_1	H→L (96%)	902/1.37	0.2574
	S_4	H − 3→L (18%); H − 2→L + 1 (14%); H − 1→L (52%); H→L + 2 (13%)	604/2.05	0.2844
	S_9	H − 2→L + 1 (41%); H − 1→L + 2 (45%)	481/2.58	0.9151
BMK	S_1	H − 1→L + 1 (13%); H→L (83%)	582/2.13	0.4536
	S_4	H − 2→L + 1 (19%); H − 1→L (29%); H→L + 1 (51%)	430/2.88	0.4875
	S_5	H − 2→L (22%); H − 1→L + 1 (58%)	403/3.08	2.2109
ωB97XD	S_1	H − 1→L + 1 (27%); H→L (66%)	595/2.08	0.2667
	S_3	H − 2→L (39%); H − 1→L + 1 (30%); H→L (20%)	428/2.90	0.6154
	S_5	H − 2→L (42%); H − 1→L + 1 (37%)	391/3.17	1.7495
HSE06	S_1	H→L (94%)	677/1.83	0.3660
	S_3	H − 2→L (78%); H − 1→L + 1 (21%)	537/2.31	0.1294
	S_7	H − 2→L (11%); H − 1→L + 1 (39%); H→L + 2 (21%)	423/2.93	1.1253
LC-ωPBE	S_1	H − 1→L + 1 (38%); H→L (59%)	628/1.98	0.1397
	S_3	H − 1→L + 1 (48%); H→L (36%)	399/3.11	1.7768
OPT-LC-ωPBE	S_1	H→L (96%)	874/1.42	0.2542
	S_4	H − 3→L (19%); H − 2→L + 1 (15%); H − 1→L (62%)	598/2.07	0.3024
	S_9	H − 2→L + 1 (50%); H − 1→L + 2 (31%)	475/2.61	1.1893
MPW1K	S_1	H − 1→L + 1 (20%); H→L (75%)	575/2.16	0.3731
	S_3	H − 2→L (62%); H − 1→L + 1 (21%); H→L (11%)	461/2.69	0.1178
	S_5	H − 2→L (29%); H − 1→L + 1 (51%); H→L (10%)	401/3.09	2.2289

Table S4. The different functional calculated excitation energies (eV), wavelength (nm), oscillator strengths (f) and major transition configurations with coefficients larger than 10% of three stronger absorption bands in UV-vis region for YD2-o-C8 in THF.

Functional	States	Major transition configurations	E(nm/eV)	*f*
CAM-B3LYP	S_1	H − 1→L + 1 (23%); H→L (73%)	592/2.10	0.3470
	S_3	H − 2→L (53%); H − 1→L + 1 (25%); H→L (12%)	439/2.82	0.3594
	S_5	H − 2→L (33%); H − 1→L + 1 (45%); H→L (11%)	397/3.12	1.8676
M062X	S_1	H − 1→L + 1 (19%); H→L (78%)	579/2.14	0.4027
	S_3	H − 2→L (61%); H − 1→L + 1 (23%)	447/2.78	0.1888
	S_5	H − 2→L (28%); H − 1→L + 1 (52%); H→L (11%)	397/3.12	1.9965
PBE0	S_1	H→L (94%)	879/1.41	0.2944
	S_3	H − 3→L (17%); H − 1→L (68%)	619/2.00	0.2027
	S_{16}	H − 7→L + 1 (15%); H − 2→L + 1 (25%); H − 1→L + 2 (35%)	477/2.60	0.7692
HSE06	S_1	H→L (93%)	671/1.85	0.4237
	S_3	H − 2→L (78%); H − 1→L + 1 (17%)	535/2.32	0.1058
	S_9	H − 1→L + 1 (42%); H→L + 2 (25%)	420/2.95	1.0332
LC-ωPBE	S_1	H − 1→L + 1 (35%); H→L (63%)	632/1.96	0.1792
	S_3	H − 2→L (11%); H − 1→L + 1 (51%); H→L (32%)	399/3.11	1.7253
OPT-LC-ωPBE	S_1	H→L (95%)	853/1.45	0.2862
	S_3	H − 3→L (25%); H − 1→L (63%)	614/2.02	0.1651
	S_{16}	H − 2→L + 1 (35%); H − 1→L + 2 (29%)	472/2.63	1.0264

Figure S1. Plot of the error function $\delta_{IP}(\omega)$ used for optimization of ω in the long-range corrected DFT functionals.

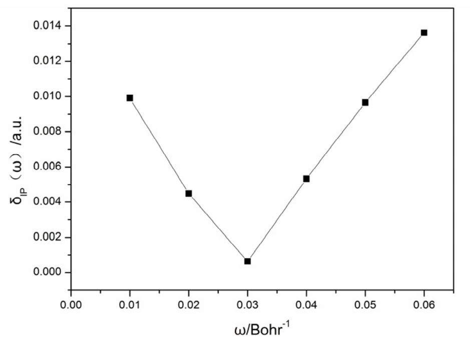

YD2

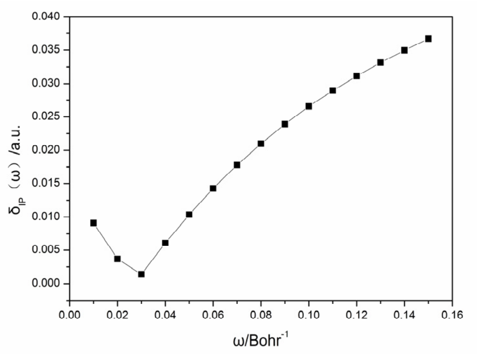

YD2-o-C8

© 2013 by the authors; licensee MDPI, Basel, Switzerland. This article is an open access article distributed under the terms and conditions of the Creative Commons Attribution license (http://creativecommons.org/licenses/by/3.0/).
